# Factors associated with Health-Related Quality of Life in Kidney Transplant Recipients in France

**DOI:** 10.1186/s12882-018-0893-6

**Published:** 2018-04-27

**Authors:** Yosra Mouelhi, Elisabeth Jouve, Marine Alessandrini, Nathalie Pedinielli, Valérie Moal, Aurélie Meurette, Elisabeth Cassuto, Georges Mourad, Antoine Durrbach, Bertrand Dussol, Stéphanie Gentile

**Affiliations:** 1 Laboratoire de Santé Publique, Faculté de Médecine, Université Aix-Marseille, 3279 Marseille, EA France; 2 Service Santé Publique et Information Médicale, CHU Marseille, Marseille, France; 3 Centre de Néphrologie et de Transplantation Rénale, CHU Marseille, Marseille, France; 4 Transplantation, Urology and Nephrology Institute (ITUN), CHU Nantes, Nantes, France; 5 Hôpital Pasteur, Nice, France; 6 Département de Néphrologie, Dialyse et Transplantation, CHU Montpellier, Montpellier, France; 7 Service de Néphrologie, CHU Bicêtre, Paris, France

**Keywords:** Cross sectional, Health-related quality of life, Kidney Transplant Recipients, ReTransQol, SF-36

## Abstract

**Background:**

Health-Related Quality of Life (HRQoL) assessment after kidney transplantation has become an important tool in evaluating outcomes. This study aims to identify the associated factors with HRQoL among a representative sample size of Kidney Transplant Recipients (KTR) at the time of their inclusion in the study.

**Methods:**

Data of this cross-sectional design is retrieved from a longitudinal study conducted in five French kidney transplant centers in 2011, and included KTR aged 18 years with a functioning graft for at least 1 year. Measures include demographic, psycho-social and clinical characteristics. To evaluate HRQoL, the Short Form-36 Health Survey (SF-36) and a HRQoL instrument for KTR (ReTransQol) were administered. Multivariate linear regression models were performed.

**Results:**

A total of 1424 patients were included, with 61.4% males, and a mean age of 55.7 years (±13.1). Demographic and clinical characteristics were associated with low HRQoL scores for both questionnaires. New variables were found in our study: perceived poor social support and being treated by antidepressants were associated with low scores of Quality of Life (QoL), while internet access was associated with high QoL scores.

**Conclusion:**

The originality of our study’s findings was that psycho-social variables, particularly KTR treated by antidepressants and having felt unmet needs for any social support, have a negative effect on their QoL. It may be useful to organize a psychological support specifically adapted for patients after kidney transplantation.

## Background

In public health and medicine, Health-Related Quality of Life (HRQoL) measurements have become an important outcome measure in addition to morbidity and mortality rates, both in population health assessment and in clinical trials [1, 2]. HRQoL is a multi-dimensional concept that includes domains related to physical, mental, emotional, and social functioning. It goes beyond direct measures of population health, life expectancy and causes of death, and focuses on the impact that health status has on Quality of Life (QoL) [2]. In addition to its multidimensional nature, one important reason to measure the HRQoL is establishing and expanding information about the range of problems that affect the patients [3–5].

In general, chronic diseases are increasingly widespread [6]. The World Health Organization (WHO), therefore, prioritizes HRQoL improvement for people living with chronic diseases [7]. In France, the August 9, 2004 public health law applied this priority, implementing a national plan to improve the HRQoL for people living with chronic diseases, mainly those with End-Stage Renal Disease (ESRD) [8]. The French Biomedicine Agency and the National Institute for Public Health Surveillance have promoted studies to determine the level of HRQoL of ESRD patients in France in order to improve the HRQoL of chronic disease patients [9, 10].

When compared with dialysis, renal transplantation has become the most cost-effective treatment [11–13] for ESRD patients, as it presents medical costs reduction, extended lifetime [14] and enhanced HRQoL [11, 15, 16].

Although the HRQoL advantages in Kidney Transplant Recipients (KTR) were established [11, 14, 16–21], life after kideny transplantation may present negative as well as positive aspects. Therefore, it is essential to describe the demographic and clinical factors that influence HRQoL outcomes. Furthermore, most published studies did not clearly explore the psycho-social variables that potentially can affect the QoL. This study aims to identify factors associated with HRQoL through a comprehensive analysis of demographic, psycho-social and clinical characteristics among a representative cohort of KTR living in France.

## Methods

### Study design and patients

This is a cross-sectional design retrieved from a longitudinal study carried out continuously during the year 2011, in five French kidney transplant centers: Marseille, Montpellier, Nice, Paris and Nantes University Hospitals. All patients aged 18 years and older with a functioning graft for at least one year were eligible for the study. Multi-organ transplant patients before or simultaneously with their kidney transplant were excluded.

### Data collection and measures

Patients were included in 2011 during their regular medical visits. Data of inclusion, including demographic, psycho-social characteristics and HRQoL, were directly collected from the patients who agreed to participate, except health data which were obtained from nephrologists.

#### Demographic and psycho-social characteristics

Demographic and psycho-social variables collected were:

➢ Age, gender, level of education: primary or less, college, secondary 1st stage and university

➢ Living arrangement: alone or not alone

➢ Having children or not, employment status: employed, retired, unemployed

➢ Disability pension: patients receiving disability pension or not

➢ Monthly incomes in the household (€)

➢ Internet and social network use

➢ Social support: done by a perceived questionnaire, used to estimate the availability and the quality of this support [22]. It is composed of four main scales: esteem, financial, informative and emotional supports. For each scale, patients were asked to answer if they were in need for this support or not.

#### Clinical characteristics

Medical measures were grouped into four domains related to kidney disease, health status and comorbidities, treatments (i.e. drugs) along with their side effects and biological data.

1. To explore kidney disease: we collected the etiology of End-Stage Renal Disease (ESRD), the previous dialysis treatment and duration, the duration since transplantation, the organ donor type (cardiac death, deceased or living-related donor), the organ transplantation (one or two simultaneous kidney grafts), the number of transplantations, the graft rejection episodes and the graft chronic dysfunction.
2. To explore health status and comorbidities: we collected the pathologies frequently associated with KTR (neoplasia, hypertension, and diabetes mellitus), smoking status, Body Mass Index (BMI) and the two validated scales: The Karnofsky Performance Scale (KPS) and The Charlson Comorbidity Index (CCI).

The Karnofsky Performance Scale (KPS) was evaluated to classify patients according to their functional impairment from 0 to 100%. The lower the Karnofsky score, the worse the survival for most serious illnesses was [23, 24].

The Charlson Comorbidity Index (CCI) was evaluated to classify patient’s comorbidities. According to Charlson et al. [25], the CCI was calculated by assigning for each pathology a score of 1, 2, 3, or 6, depending on the death risk associated with each one and by summing the weights for all present comorbid conditions (ranges from 0 to 37). For the combined age-comorbidity score, each decade of age over 40 adds 1 point to the risk (e.g. 50–59 years, 1 point; 60–69 years, 2 points; 70–79 years, 3 points…) [26]. Higher scores indicate greater comorbidity.

3. To explore treatments and their side effects, we collected treatment characteristics. We generated with nephrologists a selective list of the most commonly prescribed drugs with their generic names, previously used in a recent publication [27]. This list includes the most used categories of treatments by KTR: immunosuppressive, antihypertensive and other treatments. For each category, we obtained its corresponding drugs. Then, we asked the patients for the existence of side effects related to treatments, without specifying the type.
4. To explore biological data: we collected creatinine and hemoglobin (Hb) levels directly from nephrologists during the medical visit. Creatinine levels were defined by establishing 3 categories with nephrologists (normal < 120 μmol/L, mild to moderate: 120–250 μmol/L, severe > 250 μmol/L). Anemia was defined by using the World Health Organization (WHO) criteria, which meant that the Hb concentration was below 12 g/dl in women and below 13 g/dl in men [28].

#### Health-related quality of life

HRQoL was measured with the SF-36 and the ReTransQol. French version of the SF-36 [29, 30] is a generic, self-administered, multidimensional and coherent measure of HRQoL that consists of 36 items, which are used to calculate eight subscales: Physical Functioning (PF), Role Physical (RP), Bodily Pain (BP), General Health (GH), Vitality (VT), Social Functioning (SF), Role Emotional (RE), and Mental Health (MH). The correlated physical (PCS) and mental (MCS) summary components were computed following the standardized procedure provided by authors [27, 31, 32].

The ReTransQol version 2 [33, 34] is a disease specific self-administered instrument assessing the HRQoL of KTR and consisting of 32 items describing 5 dimensions: Physical Health (PH), Mental Health (MH), Medical Care and satisfaction (MC), Treatment (TRT), and Fear of losing the Graft (FG).

Scores for both instruments range from 0 to 100, with higher scores indicating better HRQoL.

### Ethical aspects

The study methodology was approved by the local Institutional Review Board (CCTIRS n°12-726) and the “Comité National Informatique et Liberté” (CNIL n°1639707), thus ensuring the confidentiality of all the collected informations. All patients agreeing to participate signed a written informed consent before their inclusion in the study.

### Statistical analysis

Statistical analysis was performed using Statistical Package for Social Sciences (SPSS) software (version 20, SPSS, Inc., Chicago, IL, USA). Quantitative data were expressed as mean ± standard deviation (SD), minimum and maximum or median, 25th and 75th percentiles, whereas categorical data were expressed as frequency and percentage. Group comparisons were performed using analysis of variance (bivariate analysis) for quantitative variables. All factors with a *p*-value < 0.2 were included as candidate variables in the multivariate analysis. Multivariate Linear Regression models (MLR) were used to estimate the relationship between HRQoL scores and the other characteristics. The β coefficients and p-value were performed. The level of significance was set at a p-value ≤0.05. The assumptions of the MLR were verified for linear relationship, normality of distribution, absence of multicollinearity and residuals. Because missing data were minimal (< 10%), we did not replace them and we only analyzed the available ones (i.e. ignoring the missing data).

## Results

At the time of the survey, 1469 KTR met eligible criteria for the study during 2011. Among them, 45 patients (3.1%) without QoL questionnaires were excluded from this study and 1424 were included and selected for analysis. Thus, the participation rate is 96.9%.

### Patients’ characteristics

As presented in Table 1, patient’s mean age was 55.7 years (± 13.1), more than 60% were males and lived with a partner. Less than 40% attended college and were employed at the time of the survey. The majority of KTR had access to internet in the household. Moreover, most of KTR declared having felt a need for an esteem support more than the other supports.

**Table 1:** Patients’ characteristics (*N* = 1424)

	N (%)
Gender	
Male	874 (61.4)
Age (years)	
Mean ± SD	55.7 ± 13.1
Range	18.8–85.9
Level of education	
Primary or less	163 (11.5)
College	536 (37.9)
Secondary 1st stage	304 (21.5)
University	413 (29.1)
Living arrangement	
Alone	323 (22.7)
Children	
No children	390 (27.6)
Employment status	
Employed	548 (38.5)
Retired	545 (38.3)
Unemployed	329 (23.2)
Patients receiving disability pension	487 (34.7)
Monthly incomes in the household (€)	
< 739	100 (7.5)
740–1200	255 (19.1)
1201–2200	419 (31.4)
2201–4400	425 (31.8)
> 4400	136 (10.2)
Internet	
Patients with Internet use	1131 (79.9)
Patients with Social networks use	417 (36.9)
Perceived social support	
Patients in need for an esteem support	869 (61.1)
Patients in need for a financial support	377 (26.5)
Patients in need for an informative support	559 (39.4)
Patients in need for an emotional support	592 (41.8)

*SD* standard deviation

Regarding the clinical characteristics (Table 2), more than a third of KTR had glomerulonephritis and the majority were dialyzed before transplantation. Median time since transplantation was 7.1 years. Most of patients had a deceased donor transplantation and had a single kidney transplantation. The mean CCI score was 4.09 (± 1.8, range from 2 to 14), and most of KTR had mild to moderate creatinine level (50.8%). Other characteristics about clinical characteristics are shown in Table 2. Most patients were treated with immunosuppressive drugs. Nearly 28% of KTR reported side effects related to treatments with a mean number of side effects of 6.4 ± 1.7 per patient (Table 3).

**Table 2:** Clinical characteristics: kidney disease, health status, comorbidities and biological data

	N (%)
Major causes of ESRD	
Chronic glomerulonephritis	487 (34.3)
Interstitial nephropathy	158 (11.3)
Polycystic kidney disease	262 (18.8)
Other nephropathies (vascular, diabetic…)	497 (35.6)
Previous dialysis treatment	
Patient with dialysis treatment	1212 (86.8)
Duration of dialysis, Median (25th percentiles, 75th percentiles)	24 (12, 42)
Transplantation	
Duration of transplantation, Median (25th percentiles, 75th percentiles)	7.1 (3.7, 12.8)
Organ donor type	
Cardiac death donor	47 (3.4)
Deceased donor	1231 (88.3)
Living-related donor	116 (8.3)
Organ transplantation	1396 (98.0)
Only one kidney graft	1373 (98.4)
Two simultaneously kidney grafts	23 (1.6)
Kidney Transplants number	
The first transplant	1199 (85.7)
The second transplants	181 (12.9)
Three or more transplants	19 (1.4)
Patients with at least one acute rejection episode	213 (15.3)
Patients with chronic graft dysfunction	426 (30.6)
Comorbidities	
Neoplasia	285 (20.4)
Hypertension	1143 (81.8)
Diabetes mellitus	255 (18.2)
Smoking patients	203 (14.9)
BMI > 30 (kg/m^2^)	213 (15.3)
Karnofsky Index scale, rating criteria (%)	
80–100: Able to carry on normal activity and to work	1311 (94.2)
50–70: Unable to work	76 (5.5)
20–40: Unable to care for self	3 (0.2)
0–10: Death, disease may be progressing rapidly	2 (0.1)
Charlson Comorbidity Index, score	
Mean ± SD	4.09 ± 1.8
Range	2–14
Biological data	
Creatinine levels (μmol/L)	
Normal: < 120	583 (42.6)
Mild to moderate: 120–250	697 (50.8)
Severe: > 250	91 (6.6)
Hemoglobin levels (g/dl)	
Anemia: < 12	551 (40.4)
Normal: hemoglobin ≥12	813 (59.6)

*ESRD* End-Stage Renal Disease, *SD* standard deviation

**Table 3:** Clinical characteristics: treatments and their side effects

	Drugs N (%)	Side effects^a^ N (%)
Mean number of drugs/side effects per patient	6.6 ± 1.8 (2–14)	6.4 ± 1.7 (4–9)
Immunosuppressive treatments	1397 (98.1)	344 (24.6)
Calcineurin inhibitors	1170 (82.2)	242 (20.6)
Mycophenolic acid and derivatives	933 (65.5)	115 (12.3)
Corticoids	832 (58.4)	159 (19.1)
Others (mTOR inhibitors, Azathioprine)	334 (23.4)	56 (16.7)
Antihypertensive treatments	1161 (81.5)	72 (6.2)
Beta-blockers	727 (51.1)	28 (3.8)
Angiotensin-converting enzyme (ACE) inhibitors	709 (49.8)	34 (4.8)
Calcium antagonists	485 (34.1)	20 (4.1)
Others (Central antihypertensive, Peripheral vasodilators/alpha-blockers, Diuretics)	544 (38.2)	30 (5.5)
Other treatments	1170 (82.2)	54 (4.6)
Cardiovascular drugs	792 (55.6)	38 (48.0)
Calcium drugs	727 (51.1)	2 (0.3)
Diabetes drugs	239 (16.8)	13 (5,4)
Erythropoiesis-Stimulating Agents (ESA)	199 (14.0)	1 (0.5)
Antidepressants	149 (10.5)	4 (2.7)

^a^Percentage of side effects = number of patients with a side effect related to the treatment dividing by number of patients taking this treatment

#### Health related quality of life

**Fig. 1:**
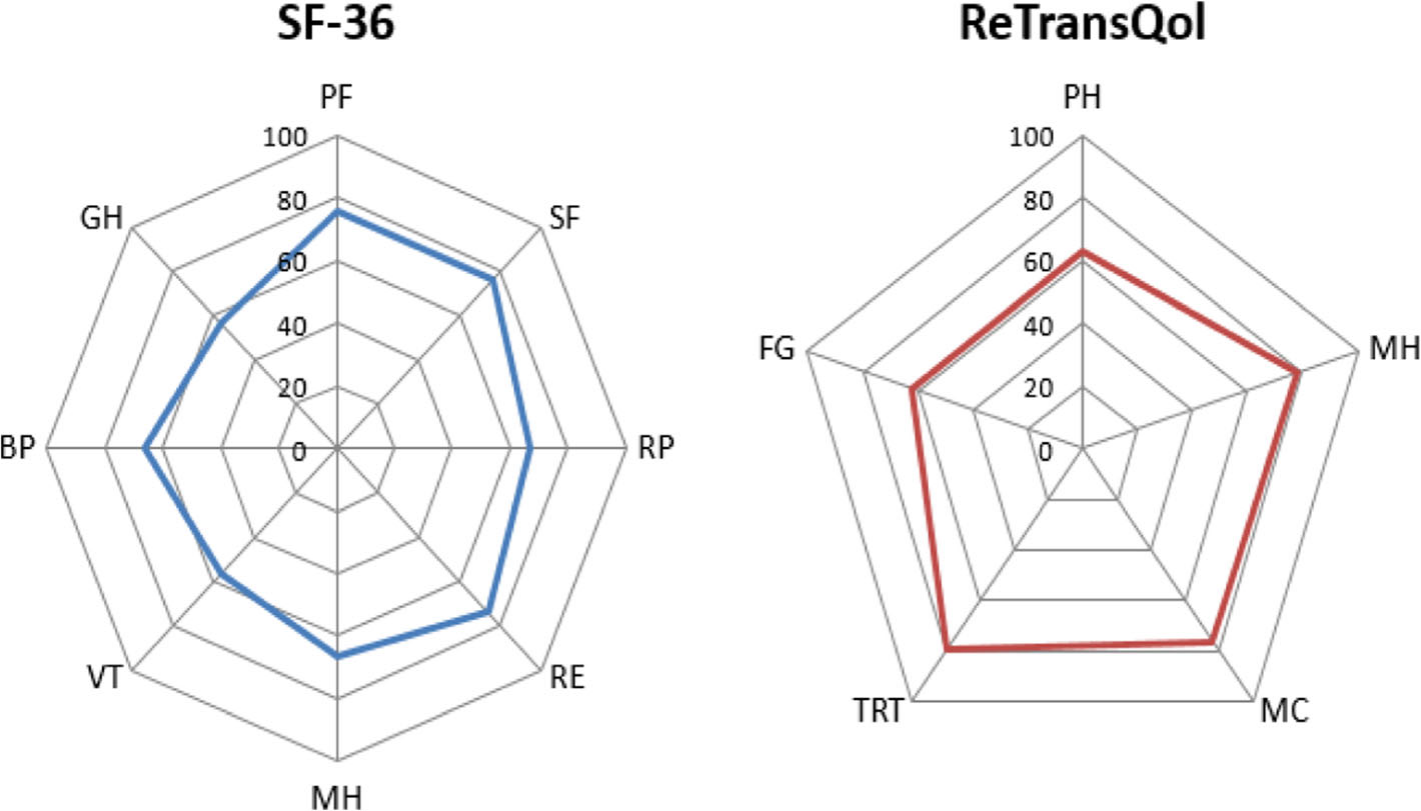
HRQoL scores (SF36 & ReTransQol)

Figure 1 shows the mean HRQoL scores and their SD for the eight components of the SF-36 and the five components of the ReTransQol.

### Multivariate regression analysis

We selected all variables in the final regression model for both questionnaires (SF-36 and ReTransQol) according to their significance in the univariate analysis (*p* < 0.2).

Adjusted differences in the ten generic dimensions of SF-36 and in the five specific dimensions of ReTransQol using demographic, psycho-social and clinical variables are shown in Tables 4 and 5, respectively.

**Table 4:** Final regression models for SF-36 domains

Dimensions	Variables	β coeff. [95% CI]	*P* values
PF *N = 1253* *R*^*2*^ *= 0.21*	Intercept	84.1 [75.3; 92.8]	< 0.001
	Age	−0.3 [− 0.4; − 0.1]	< 0.001
	Female	−4.4 [−7.0; −1.8]	0.001
	Disability pension	−6.3 [−9.0; −3.7]	< 0.001
	High monthly incomes (€)	3.5 [2.3; 4.7]	< 0.001
	Having felt a need for an esteem support	−4.1 [− 6.8; − 1.3]	0.004
	Having felt a need for a financial support	−3.1 [− 6.1; − 0.1]	0.04
	Diabetic patient	− 4.5 [− 7.9; − 1.2]	0.008
	BMI > 30 (kg/m^2^)	− 4.0 [− 7.4; − 0.6]	0.021
	Treatment with ESA	− 3.7 [− 7.3; − 0.1]	0.045
	Treatment with antidepressants	−5.7 [− 9.7; − 1.7]	0.005
	KPS ≥ 70%	16.9 [11.6; 22.2]	< 0.001
	High creatinine levels > 250 (μmol/L)	− 2.7 [− 4.8; − 0.6]	0.012
	CCI score	− 1.4 [− 2.4; − 0.4]	0.005
RP *N = 1256* *R*^*2*^ *= 0.14*	Intercept	84.0 [69.0; 99.0]	< 0.001
	Age	−0.4 [− 0.5; − 0.2]	< 0.001
	Disability pension	− 7.4 [− 12.0; − 2.7]	0.002
	High monthly incomes (€)	4.5 [2.5; 6.5]	< 0.001
	Having felt a need for an esteem support	−6.4 [− 11.5; − 1.3]	0.015
	Having felt a need for an emotional support	−8.4 [−13.6; − 3.3]	0.001
	Diabetic patient	−6.2 [−11.7; − 0.8]	0.026
	Treatment with ESA	−8.9 [−15.0; −2.7]	0.005
	Treatment with antidepressants	−15.2 [− 22.2; − 8.2]	< 0.001
	KPS ≥ 70%	14.6 [5.5; 23.6]	0.002
	High creatinine levels > 250 (μmol/L)	−5.9 [−9.4; − 2.4]	0.001
BP *N = 1257* *R*^*2*^ *= 0.15*	Intercept	85.3 [74.7; 95.9]	< 0.001
	Age	−0.3 [− 0.4; − 0.2]	< 0.001
	Female	−4.6 [−7.7; −1.5]	0.003
	Having children	−3.9 [− 7.4; − 0.4]	0.03
	Disability pension	−6.3 [−9.5; −3.1]	< 0.001
	High monthly incomes (€)	1.8 [1.4; 3.3]	0.014
	Having felt a need for an esteem support	−5.2 [−8.5; −1.9]	0.002
	Diabetic patient	−6.0 [−9.8; −2.2]	0.002
	Treatment with cardiovascular drugs	−3.8 [−6.9; −0.7]	0.017
	Treatment with antidepressants	−9.6 [− 14.4; −4.7]	< 0.001
	Side effects related to any treatment	−5.8 [− 9.0; − 2.5]	< 0.001
	KPS ≥ 70%	14.2 [7.9; 20.5]	< 0.001
	CCI score	−2.7 [−5.2; −0.3]	0.030
GH *N = 1321* *R*^*2*^ *= 0.15*	Intercept	63.9 [58.4; 69.4]	< 0.001
	Living alone	−3.8 [−6.4; −1.2]	0.004
	Disability pension	−6.0[−8.3; − 3.7]	< 0.001
	Having felt a need for an esteem support	−4.3 [− 6.9; − 1.6]	0.002
	Having felt a need for an emotional support	−3.2[−5.9; − 0.6]	0.016
	Duration of transplantation	−0.2 [− 0.3; − 0.0]	0.03
	Treatment with cardiovascular drugs	−4.9 [− 7.1; − 2.7]	< 0.001
	Treatment with ESA	− 4.9 [−8.1; − 1.7]	0.003
	Treatment with antidepressants	− 4.3 [− 7.9; − 0.7]	0.018
	KPS ≥ 70%	10.0 [5.3; 14.8]	< 0.001
	High creatinine levels > 250 (μmol/L)	−6.9 [−8.7; − 5.0]	< 0.001
SF *N = 1362* *R*^*2*^ *= 0.16*	Intercept	86.6 [79.6; 93.7]	< 0.001
	Living alone	−5.1 [−8.0; −2.1]	0.001
	Disability pension	−2.6 [−5.3; −0.0]	0.05
	Having felt a need for an esteem support	−4.1 [− 7.3; − 0.9]	0.011
	Having felt a need for an emotional support	−7.8 [−10.9; − 4.8]	< 0.001
	Treatment with ESA	−8.1 [−11.8; − 4.5]	< 0.001
	Treatment with calcium drugs	−3.9 [−6.4; − 1.4]	0.002
	Treatment with antidepressants	−12.4 [− 16.5; − 8.3]	< 0.001
	KPS ≥ 70%	8.4 [2.9; 13.8]	0.003
	CCI score	−0.9 [−1.6; − 0.2]	0.01
RE *N = 1257* *R*^*2*^ *= 0.15*	Intercept	72.5 [62.4; 82.6]	< 0.001
	Disability pension	−6.4 [−10.7; − 2.2]	0.003
	High monthly incomes (€)	3.4 [1.5; 5.3]	< 0.001
	Having felt a need for an esteem support	−7.0 [−11.8; − 2.2]	0.004
	Having felt a need for an emotional support	−5.9 [−9.4; − 2.4]	< 0.001
	Treatment with antidepressants	−14.4 [− 25.8; − 12.9]	< 0.001
	KPS ≥ 70%	13.3 [5.0; 21.6]	0.002
	High creatinine levels > 250 (μmol/L)	−4.4 [−7.7; − 1.1]	0.009
MH *N = 1278* *R*^*2*^ *= 0.18*	Intercept	60.6 [55.7; 65.5]	0.000
	Disability pension	−5.9 [−9.4; −2.4]	0.020
	High monthly incomes (€)	2.7 [1.8; 3.7]	< 0.001
	Having felt a need for an esteem support	−6.1 [−8.7; − 3.6]	< 0.001
	Having felt a need for an emotional support	−5.1 [− 7.6; −2.6]	< 0.001
	Treatment with antidepressants	−15.2 [− 18.4; − 11.9]	< 0.001
	KPS ≥ 70%	7.8 [3.6; 12.0]	< 0.001
VT *N = 1257* *R*^*2*^ *= 0.15*	Intercept	59.2 [52.6; 65.7]	< 0.001
	High monthly incomes (€)	2.3 [1.3; 3.3]	< 0.001
	Having felt a need for an esteem support	−4.4 [−7.0; −1.8]	0.001
	Having felt a need for an emotional support	−3.7 [−6.2; − 1.2]	0.004
	Treatment with ESA	−3.4 [−6.5; −0.3]	0.03
	Treatment with calcium drugs	−4.4 [−6.5; −2.3]	< 0.001
	Treatment with antidepressants	−8.8 [−12.3; −5.4]	< 0.001
	KPS ≥ 70%	8.0 [3.4; 12.5]	0.001
	High creatinine levels > 250 (μmol/L)	−2.8 [−4.6; −1.0]	0.002
	CCI score	−0.8 [−1.4; − 0.2]	0.007
PCS *N = 1244* *R*^*2*^ *= 0.2*	Intercept	51.2 [47.4; 54.8]	< 0.001
	Age	−0.1 [− 0.1; − 0.0]	0.003
	Female	−1.8 [−2.8; − 0.7]	0.001
	Disability pension	−2.7 [−3.8; − 1.6]	< 0.001
	High monthly incomes (€)	−0.9 [− 0.4; − 1.3]	< 0.001
	Treatment with diabetic drugs	− 2.5 [−3.9; − 1.1]	< 0.001
	Treatment with cardiovascular drugs	− 1.4 [− 2.4; − 0.3]	0.009
	Side effects related to any treatment	−1.4 [− 2.5; − 0.3]	0.012
	KPS ≥ 70%	5.9 [3.7; 8.0]	< 0.001
	High creatinine levels > 250 (μmol/L)	−2.1 [− 3.0; − 1.3]	< 0.001
	CCI score	− 0.7 [− 1.1; − 0.3]	0.001
MCS *N = 1268* *R*^*2*^ *= 0.18*	Intercept	49.6 [47.9; 51.2]	< 0.001
	High monthly incomes (€)	1.3 [0.8; 1.8]	< 0.001
	Having felt a need for an esteem support	−2.8 [−4.1; −1.4]	< 0.001
	Having felt a need for an emotional support	−4.0 [−5.4; − 2.7]	< 0.001
	Treatment with calcium drugs	−1.1 [−2.2; −0.0]	0.044
	Treatment with antidepressants	−7.3 [−9.1; −5.5]	< 0.001
	High creatinine levels > 250 (μmol/L)	−1.1 [−2.0; −0.2]	0.015

*β coeff* β coefficient, *ESA* Erythropoiesis-Stimulating Agent, *KPS* Karnofsky Performance Scale, *CCI* Charlson Comorbidity Index, *PF* Physical Functioning, *RP* Role Physical, *BP* Bodily Pain, *GH* General Health, *VT* Vitality, *SF* Social Functioning, *RE* Role Emotional, *MH* Mental Health, *PCS* Physical Component Score, *MCS* Mental Component Score

**Table 5:** Final regression models for ReTransQol domains

Dimensions	Variables	β coeff. [95% CI]	*P* values
PH *N = 1340* *R*^*2*^ *= 0.1*	Intercept	65.2 [61.3; 69.0]	< 0.001
	Employment status	2.1 [0.6; 3.5]	0.005
	Disability pension	−2.8 [−4.2; −1.5]	< 0.001
	Having felt a need for an esteem support	−3.1 [−4.6; −1.6]	< 0.001
	Having felt a need for an emotional support	−1.7 [− 3.3; −0.2]	0.022
	Treatment with cardiovascular drugs	−1.7 [−3.0; −0.4]	0.009
	Treatment with antidepressants	−4.2 [−6.2; −2.1]	< 0.001
	Side effects related to any treatment	−1.4 [−2.8; −0.07]	0.04
	KPS ≥ 70%	5.4 [2.6; 8.1]	< 0.001
	High creatinine levels > 250 (μmol/L)	−1.5 [−2.6; −0.5]	0.003
MH *N = 1276* *R*^*2*^ *= 0.1*	Intercept	77.5 [73.7; 81.4]	< 0.001
	Female	−2.0 [−3.8; −0.1]	0.034
	Living alone	−5.1 [−7.3; −3.0]	< 0.001
	Disability pension	−2.3 [−4.3; −0.4]	0.015
	High monthly incomes (€)	1.4 [0.5; 2.4]	0.002
	Internet use	5.4 [0.2; 5.0]	0.03
	Having felt a need for an informative support	2.1 [0.2; 3.8]	0.024
	Treatment with antidepressants	−7.0 [−9.8; −4.0]	< 0.001
MC *N = 1355* *R*^*2*^ *= 0.1*	Intercept	78.2 [76.9; 79.6]	< 0.001
	Having felt a need for an informative support	1.9 [0.5; 3.4]	0.008
	Treatment with ESA	−7.0 [−9.8; −4.0]	0.06
	Treatment with calcium drugs	−2.3 [−3.7; −0.8]	0.001
	High creatinine levels > 250 (μmol/L)	− 2.0 [− 3.1; − 0.7]	0.002
TRT *N = 1347* *R*^*2*^ *= 0.1*	Intercept	86.8 [82.2; 91.5]	< 0.001
	High educational level	−3.4 [−6.1; −0.7]	0.011
	Having felt a need for an esteem support	−4.2 [− 6.3; −2.1]	< 0.001
	Having felt a need for an informative support	−3.1 [−5.2; −1.1]	0.003
	Treatment with calcium drugs	−2.2 [−4.0; −0.5]	0.011
	Side effects related to any treatment	−2.2 [−4.1; −0.3]	0.023
	KPS ≥ 70%	4.1 [0.4; 7.7]	0.026
	High creatinine levels > 250 (μmol/L)	−3.1 [−4.5; −1.8]	< 0.001
FG *N = 1259* *R*^*2*^ *= 0.1*	Intercept	70.4 [66.1; 74.4]	< 0.001
	Disability pension	−4.0[−6.2; −1.7]	0.001
	High monthly incomes (€)	1.9 [0.4; 2.9]	< 0.001
	Having felt a need for an esteem support	−4.1 [−6.7; −1.6]	0.001
	Having felt a need for an emotional support	−3.7 [−6.3; −1.2]	0.004
	Being on dialysis before transplantation	−4.8 [−7.8; −1.7]	0.002
	Treatment with antidepressants	−3.6 [− 7.0; −0.2]	0.037
	High creatinine levels > 250 (μmol/L)	−3.7 [−5.9; −2.1]	< 0.001

*β coeff* β coefficient, *ESA* Erythropoiesis-Stimulating Agent, *KPS* Karnofsky Performance Scale, *PH* Physical Health, *MH* Mental Health, *MC* Medical Care and satisfaction, *TRT* Treatment, *FG* Fear of losing the Graft

All HRQoL components of SF-36 and RTQ were lower with demographic and clinical characteristics. The variables which contributed most to low QoL scores were receiving disability pension, low monthly incomes and a low Karnofsky Performance Scale (< 70%). To a lesser extent, advanced age, female gender, having children, unemployment, living alone, a high Charlson Comorbidity Index, high creatinine levels (> 250 μmol/L), chronic graft dysfunction, being treated with cardiovascular drugs and presence of side effects related to treatments were associated with low QoL scores (Tables 4 and 5).

Psycho-social variables were also found to be associated with HRQoL scores for both questionnaires. Perceived poor social support and being treated by antidepressants were associated with low scores of QoL, while internet access was associated with high QoL scores (Tables 4 and 5).

## Discussion

This study analyzed the factors associated with HRQoL in a representative sample of 1424 Kideny Transplant Recipients (KTR) from five kidney centers of France. This study goes further from a previous work published in a French national study of 1061 KTR from 8 regions of France, which was the first French report about HRQoL in kidney transplantation [27]. We analyzed the exploration of psycho-social factors that were poorly studied in literature, such as perceived social support measured by a validated questionnaire [22] and internet access. Indeed, there is growing evidence for the necessity of specifying psychological dimension’s influence on quality of life after kidney transplantation [35, 36].

In our study, HRQoL scores, socio-demographic and clinical characteristics of KTR are similar to a French national survey [27]. Socio-demographic variables had a negative influence on HRQoL: level of HRQoL significantly decreases with age, female gender, living status and the educational level. These findings are in accordance with other studies [27, 37–52].

This study points out that KTR receiving a disability pension have an extremely impaired HRQoL, especially for physical dimensions. This association may be influenced by the impact of manual work [53]. Patients in lower-ranked occupations may have less control in the work situation and thereby less possibility to prevent their health influenced by physical demands and poor ergonomic working environment [54].

Karnofsky Performance Status (KPS) was strongly associated with good HRQoL scores, whatever the instrument used. Whether KTR could carry normal activity and take care of themselves are the important parameters of measurement of QoL. Zhang L et al. [55] suggested that KPS score could be the most important factor associated with QoL values in patients with advanced HIV. These results suggest that healthy lifestyle and physical function are recommended after transplantation to improve HRQoL and it seems important to counsel and encourage for more physical activity as a part of routine medical care in KTR.

Furthermore, treatments with diabetic, cardiovascular and calcium drugs had a negative impact on HRQoL, especially for physical dimensions. In contrast, we did not find any association between immunosuppressive drugs and HRQoL, which suggests that nephrologists should use more effective treatments to prevent rejection and preserve the kidney function without adversely affecting HRQoL. A specific health education for KTR, including how treatments must be adhered, its benefits and side effects, is also recommended for KTR to handle difficulties due to specific treatments.

We also found a strong association between antidepressants and bad HRQoL scores for both physical and mental components. It is possible that patients treated with antidepressants tended to somatize more and give more emphasis to the negative effects of transplantation than its positive effects [56, 57]. It could also suggest that patients with worse health conditions are more susceptible to depression, even after transplantation. Studies reported that patients undergoing dialysis and/or transplantation, may become unable to cope with it, as it affects their mind integrity [58]. Depression and anxiety as impaired HRQoL are known to be associated with increased mortality and poor outcomes in KTR [59, 60]. Mental health is thus playing an important role in HRQoL and should not be underestimated after kidney transplantation.

Aside from these factors, social support was significantly associated with bad HRQoL scores. KTR with a need for an esteem and emotional support have bad QoL scores in almost all dimensions and specifically in mental dimensions (Tables 4 and 5). This underlies that social support may reflect non-constructive coping strategies with the disease, which should not be underestimated. Furthermore, informative support and internet access were associated with high QoL scores for mental dimension of ReTransQol (Table 5), testifying their interest in seeking for information and communication about their health or their transplant, and arguing the need for progress in these fields. These results are in accordance with previous studies, which demonstrated that Internet could improve the well-being and QoL by providing mental stimulation and challenge [61].

Summing up, these new findings reinforce the importance of patients’ psychological health and strengthen the necessity of psychosocial development and support for these patients. Our patients may, after kidney transplantation, need more psychological interventions aiming to provide information about their medical care. This could help them to deal with their disease and reduce several mental problems (such as stress and anxiety). Therefore, for better post-transplant rehabilitation and given the risks of psychopathology, the development of interdisciplinary interventions such as socio-medical and psychotherapeutic programs are essential.

Finally, our sample is representative of general French KTR with a large sample size. To our knowledge, there are few studies with a sample over 1000 patients [17, 62, 63]. Another strong point of the current study was the use of generic and specific HRQoL tools. We applied the generic instrument SF-36 Health Survey [29, 30], the most used questionnaire for HRQoL analysis in KTR [64–67], and a disease-specific instrument validated for KTR in the French language: the ReTransQol version 2 [34]. Both questionnaires are very interesting to work with, as they are complementary and offer different views on the global aspects and the specific domains to identify factors associated with HRQoL for KTR. Indeed, RTQ was more sensitive than SF-36 for clinical variables such as treatment and fear of losing the graft, but less exhaustive for demographic factors. Another strong point of this study was the construction of a comprehensive multivariate model, including many variables, especially psycho-social ones that were poorly studied in QoL studies for KTR. Despite being comprehensive, the final regression models explained 20% of the physical (PCS) HRQoL variance and 18% of mental HRQoL variance (MCS). Limitations of our research are related to the cross-sectional design, which is the first phase of our longitudinal study, so we cannot truly interpret predictive factors. The longitudinal data are currently under analysis to compare HRQoL scores, its evolution over time and its associated factors.

## Conclusion

The originality of our study’s findings was that new variables, particularly KTR treated by antidepressants and having felt unmet needs for any social support, have a negative effect on their QoL. It may be useful to organize a psychological support specifically adapted for these patients. In order to orientate psychological programs and improve patient care and well-being, a better understanding of how patients anticipate, live and face post-kidney-transplantation and a deep investigation of psychological factors are needed in future QoL studies.

## Abbreviations

BMI: Body Mass Index
BP: Bodily Pain
CCI: Charlson Comorbidity Index
ESA: Erythropoiesis-Stimulating Agents
ESRD: End-Stage Renal Disease
FG: Fear of losing the Graft
GH: General Health
Hb: Hemoglobin
HRQoL: Health-Related Quality of Life
KPS: Karnofsky Performance Scale
KTR: Kidney Transplant Recipients
MC: Medical Care and satisfaction
MCS: Mental Composite Score
MH: Mental Health
MLR: Multivariate Linear Regression
PCS: Physical Composite Score
PF: Physical Functioning
PH: Physical Health
QoL: Quality of Life
RE: Role Emotional
RP: Role Physical
RTQ: ReTransQol
SD: Standard Deviation
SF: Social Functioning
SF-36: Short Form-36 Health Survey
TRT: Treatment
VT: Vitality
WHO: World Health Organization
β coeff: β coefficient
